# Key factors controlling microbial distribution on a DNAPL source area

**DOI:** 10.1007/s11356-021-15635-2

**Published:** 2021-08-05

**Authors:** Jofre Herrero, Diana Puigserver, Ivonne Nijenhuis, Kevin Kuntze, José M. Carmona

**Affiliations:** 1 Department of Minerology, Petrology and Applied Geology, Faculty of Earth Sciences, The Water Research Institute (IdRA), University of Barcelona, C/ Martí Franquès sn, Barcelona, Spain; 2 Department of Isotope Biogeochemistry (ISOBIO), UFZ Centre for Environmental Research Leipzig-Halle, Permoserstr. 15, 04318 Leipzig, Germany; 3 Present Address: Isodetect, Deutscher Platz 5b, 04103 Leipzig, Germany

**Keywords:** T-RFLP, Toxicity, DNAPL, Microbial heterogeneity, Perchloroethene

## Abstract

Chlorinated solvents are among the common groundwater contaminants that show high complexity in their distribution in the subsoil. Microorganisms play a vital role in the natural attenuation of chlorinated solvents. Thus far, how the in situ soil microbial community responds to chlorinated solvent contamination has remained unclear. In this study, the microbial community distribution within two boreholes located in the source area of perchloroethene (PCE) was investigated via terminal restriction fragment length polymorphism (T-RFLP) and clone library analysis. Microbial data were related to the lithological and geochemical data and the concentration and isotopic composition of chloroethenes to determine the key factors controlling the distribution of the microbial communities. The results indicated that *Proteobacteria*, *Actinobacteria*, and *Firmicutes* were the most abundant phylums in the sediment. The statistical correlation with the environmental data proved that fine granulometry, oxygen tolerance, terminal electron-acceptor processes, and toxicity control microbial structure. This study improves our understanding of how the microbial community in the subsoil responds to high concentrations of chlorinated solvents.

**Supplementary Information:**

The online version contains supplementary material available at 10.1007/s11356-021-15635-2.

## Introduction

The subsoil is a heterogeneous medium with many fluctuating parameters that affect the growth and survival of microorganisms (Paul and Clark 1989). Geological factors, such as pore size and the interconnectivity of the sediments (Puigserver et al. 2020; Mahmoudi et al. 2020), as well as the biogeochemical composition of the sediments, such as the content and composition of the organic matter and the presence of metals (Van Horn et al. 2013), control the variation in the microbial communities across the subsurface environments. In saturated zones, the velocity, hydrochemical parameters, and temperature of the groundwater, as well as the composition of the planktonic microbial communities, also affect the structure of the microbial communities along the sediment (Velasco Ayuso et al. 2009; Guo et al. 2019). In pollutant episodes, the distribution of contaminants and their daughter products is controlled by geological, hydrogeological, and biogeochemical parameters (Guilbeault et al. 2005; Hartog et al. 2010; Puigserver et al. 2013), and this, in turn, conditions the microbial communities (Griebler and Lueders 2009; Schmidt et al. 2017). Specifically, the type and distribution of contaminants and their toxicity and biodegradability are key factors that explain changes in the structure of the microbial communities (Rossi et al. 2012; Puigserver et al. 2013, 2020).

Chlorinated solvents, which are types of dense non-aqueous-phase liquids (DNAPLs), are among the common groundwater contaminants that show high complexity in their distribution in the subsoil and that affect the composition of the microbial communities (Puigserver et al. 2020). Chlorinated contaminants are accidently released into the environment at many industrial and urban sites worldwide and are highly toxic (He et al. 2015). They migrate as a free phase through the porosity, are heterogeneously distributed as pools and as a residual phase between pores, and are sorbed in organic matter and fine materials due to molecular diffusion (Parker et al. 2003; Chapman and Parker 2005). Together, these migrated compounds form the source zone (Parker et al. 2003). The compounds dissolved in groundwater create large contamination plumes and can be volatilised, remaining within the gaseous matrix or dissolving again in the water (Mackay et al. 2006). The morphology of the contamination source areas is what conditions the plume of volatilised and dissolved contaminants (Pankow and Cherry 1996). In remediation strategies for chlorinated solvents, the source zone is treated in the first stage, and the plume is treated afterwards (or at the same time). Chemical and physical strategies have mostly been applied as source zone remediation strategies, rather than the biological strategies that are mainly used in the plume (Stroo 2010; Stroo et al. 2012). In recent years, however, studies have highlighted the potential for applying bioremediation strategies in source zones as well (Herrero et al. 2019; Sung and Ritalahti 2003; Yang and McCarty 2000, 2002).

Under anoxic conditions, chlorinated solvents such as chloroethenes are mainly degraded via reductive dehalogenation, which involves the sequential reduction of these compounds (Smidt and de Vos 2004). This process requires increasingly reductive conditions (Wiedemeier et al. 1999; Bradley 2003) and methanogenic conditions for their complete reductive dehalogenation to an inert compound (Hata et al. 2004). Organohalide-respiring bacteria (OHRB) are mainly responsible for reductive dehalogenation (Adrian and Löffler 2016). OHRB are usually a small percentage of the total bacterial community, compared to the fermenting and sulphate-reducing populations, which are in much greater abundance (Ndon et al. 2000; Fathepure et al. 2002; Men et al. 2012).

The complex interactions between dehalogenating microorganisms and the structure of the microbial community have been of interest during the last 2 decades (e.g. Atashgahi et al. 2017; Balaban et al. 2019; Dojka et al. 1998; Fennell et al. 2001; Flynn et al. 2000; Freeborn et al. 2005; Hendrickson et al. 2002; Hohnstock-Ashe et al. 2001; Rossi et al. 2012, 2009). In addition, a better understanding of the relationship between the structure of the microbial community and the dehalogenators will lead to the development and optimisation of bioremediation strategies. Of particular interest are the contact areas between two different geological units since shifts in microbial communities and biogeochemical processes are expected in these areas (McMahon and Chapelle 2008; Puigserver et al. 2013, 2016; Griebler and Avramov 2015).

A perchloroethene (PCE) source area in a site of alluvial fans with a highly heterogeneous geological, biogeochemical, and contaminant distribution was chosen in order to determine the main factors that condition the structure of the microbial communities in the source zone of chlorinated solvents. The goal was to identify the factors that affect the structure of the microbial communities, enabling a more detailed definition of the conceptual model of a contamination episode. The identification of these factors has the potential to improve the efficiency of bioremediation strategies. The studied factors are related to the granulometry, biogeochemical processes, and distribution of chloroethenes in the sediments and to the hydrochemistry of the aquifer. The specific objectives of the research were the following: (1) to characterise the microbial distribution of two boreholes in the source area by particularly sampling the contact areas where microbial shifts are expected and (2) to assess the main factors that affect the composition of the microbial communities.

## Materials and methods

### Site description, core sampling, and conservation protocol

The field site is in an industrial area in Vilafant (Alt Empordà, NE Spain), approximately 150 km north of Barcelona. The aquifer consists of Pliocene prograding alluvial fan deposits, and a PCE-DNAPL source was detected by the Catalan Water Agency in the transition zone to a basal aquitard.

The drilling method and the general sampling and conservation protocol are described by Puigserver et al. (2016). The core sampling was performed by taking into consideration the lithological and textural changes and by following the criteria indicated by Guilbeault et al. (2005) and Puigserver et al. (2013). A total of 60 samples were taken from the F1UB borehole (16 m depth), and 115 samples were taken from the F2UB borehole (20 m depth). Between 60 and 120 g of sediment were taken from the central part of the borehole with sterile tools. The sediment was placed inside a sterile container with distilled water and was immediately frozen to below −20°C.

A total of 29 samples, 15 from the F1UB borehole and 14 from the F2UB borehole, were selected for molecular analysis. The selection criteria were based on the detailed geological characterisation, the concentration of chloroethenes in the porewater, and the concentration of organic carbon, iron, and manganese in the sediment.

The groundwater sampling of the two multilevel wells located in the F1UB and F2UB boreholes, as well as the hydrochemical analyses, is described by Herrero et al. (2021a). Briefly, the 5 ports located in the aquifer of each multilevel well were sampled with an Eijkelkamp peristaltic pump with a Teflon pipe (with an external diameter of less than 9.5 mm) and 1 sterile 1-L glass bottle. The groundwater samples were filtered the same day in the laboratory with 0.2 μm pore size filters (Millipore, Isopore^TM^ membrane filters) and frozen to below −20°C.

### Environmental data analysis and treatment

The environmental data used in the correlation with the microbial data were the particle size of the sediment; the concentration of organic carbon, Fe, Mn, and chloroethenes; and the isotopic composition of the PCE. The content of organic carbon (Corg), Fe, and Mn sorbed in the fine fraction of sediments, as well as the chloroethene concentration analysis and calculations in the porewater, is described by Puigserver et al. (2016). The analysis of the isotopic composition of the PCE in the porewater is described by Herrero et al. (2021b).

To assess the presence or absence of the process of reductive dehalogenation, a qualitative variable was developed. It was determined that if a daughter product of PCE and/or isotopically enriched PCE in the porewater was present, reductive dehalogenation had occurred. To assess the toxicity, a new variable of the sum of all chloroethenes was used.

### Molecular analysis and data treatment

The analyses were performed at the Helmholtz Centre for Environmental Research-UFZ (Leipzig, Germany). Genomic DNA was extracted from 1.1 g of sediment with the NucleoSpin® Soil of Macherey & Nagel, following the manufacturer’s protocol, to perform terminal restriction fragment length polymorphism (T-RFLP) and clone library analysis.

Polymerase chain reaction (PCR) and clone analysis were performed according to Puigserver et al. (2016). The PCR product was purified using the Wizard® Purification Kit for Genomic DNA (Promega). A total of 50 ng of purified DNA was restricted with three different restriction enzymes (HaeIII, HhaI, and MspI, Thermo Scientific). The dry DNA was dissolved with Hi-DiTM Formamid (Applied Biosystems) with standard GeneScan™ 500 ROX™ and was analysed with an ABI 3100 Genetic Analyser (Applied Biosystems) and Genemapper 3.7 Software (Applied Biosystems). Duplicates of each sample were analysed, and consequently, six results were obtained for each sample. To validate the results, all the duplicates for each restriction enzyme were checked, and the test was repeated if the results were not conclusive.

All restriction fragments (RFs) smaller than 50 bp and having a proportion smaller than 1% of the total area were eliminated. Then, the HaeIII, HhaI, and MspI results for each sample were averaged. The microbial richness of each sample was determined by the maximum number of valid RFs for each restriction enzyme. The degree of development was determined by the average of the total area of the three restriction enzymes. Quantification of the population density using the T-RFLP technique allows for comparison of the degree of development for each of the samples. Several authors (Bruce 1997; Liu et al. 1997) recommend treating this measure as a semiquantitative value since T-RFLP analysis is subject to all the biases inherent in any PCR approach. Following these directions, the average of the areas of the different restriction enzymes was averaged again and transformed on a scale of 1 to 10.

The results obtained using T-RFLP allowed the degree of similarity to be established from a cluster analysis. Ward’s algorithm was chosen since it considers the peaks and the percentage area of each (Murtagh and Legendre 2011). This method is based on the integration of the different individuals (in this case, microbial communities) into clusters producing the minimum difference, in terms of the percentage area and the number of peaks. The method raises all possible fusions at each stage and selects the one that maximises homogeneity (or minimises heterogeneity): it calculates the centroids of the groups resulting from the possible fusions, calculates the distance to the centroid of all group observations, and chooses the solution with the smaller total quadratic sum.

The correlation between the most abundant and common RFs for the different restriction enzymes was determined by statistical treatment. Next, the phylogenetic assignment tool (PAT) (Kent et al. 2003) was used to assign potential taxa to each set of three RF signals. The PAT database was amplified with the data from the in silico project (Bikandi et al. 2004). The reported data of RFs are the means of the results for the three restriction enzymes.

### Conceptual model

The PCE source zone has been characterised by Puigserver et al. (2016) as having five hydrostratigraphic units (the unsaturated zone [UZ], the discontinuous confining aquitard [UDTA], the upper aquifer [UPA], the transition zone to the basal aquitard [TZBA], and the basal aquitard [BA]). In addition, the distribution of chloroethenes, Fe, Mn, and Corg and the richness of microbial communities have been characterised (Puigserver et al. 2016). Herrero et al. (2021b) presented a new compound-specific isotope analysis (CSIA) method for chlorinated solvents in porewater applied to the three saturated hydrostratigraphic units (UPA, TZBA, and BA).

The UZ is composed of coarse, medium, and fine gravel and sand, with a silty-clayey matrix exceeding 50%. A heterogeneous distribution of PCE was detected in this unit, with a pronounced maximum of 10,385 μg/L in the porewater in the F1UB borehole and evidence of dehalogenation above this. The UDTA consists mainly of clays, with a network of subvertical microfractures. Increased PCE was detected in the porewater in comparison with the upper unit (UZ) and the lower unit (UPA), with concentrations around 300 μg/L. The UPA is composed of gravel and coarse sand, with about 15% of the levels having a silty-clayey matrix. PCE concentrations in this zone were low, with the exception of one silty-clayey matrix level, which showed a concentration of 1150 μg/L in the porewater in the F1UB borehole, and there was evidence of dehalogenation (enriched δ^13^C_PCE_ in the porewater). The groundwater presented oxic conditions, with dissolved oxygen concentrations of around 10 mg/L. There was also evidence of denitrification, Mn reduction, and reductive dehalogenation in the upper part of the UPA (Herrero et al. 2021a). The TZBA is made up of gravel and coarse sand alternating with numerous layers of medium to fine sand and silt on a centimetre to decimetre scale of limited horizontal extension. The F1UB borehole had more levels with a clayey-silty matrix (about 90%) than the F2UB borehole, which had about 30%. A residual pool of PCE was found in the TZBA contact with the BA, with maximum concentrations in the porewater of 18,175 and 6,409 μg/L of PCE in F1UB and F2UB, respectively. Reductive dehalogenation was found to be active above this maximum PCE. The groundwater presents more reductive redox conditions as it becomes deeper, and denitrification, Mn and Fe reduction, sulphate reduction, and reductive dehalogenation of PCE and trichloroethene (TCE) were detected. No anaerobic processes were detected in the upper zone of the TZBA, and the conditions remained oxic, with a concentration of dissolved oxygen around 8 mg/L (Herrero et al. 2021a). The BA is composed of fine sands and laminar silts that are microfractured (with subvertical fractures). The distribution of PCE and TCE within the BA is ruled by the presence of vertical microfractures and stratification planes in the very fine sands with a silty-clayey matrix (Herrero et al. 2021b).

**Fig. 1:**
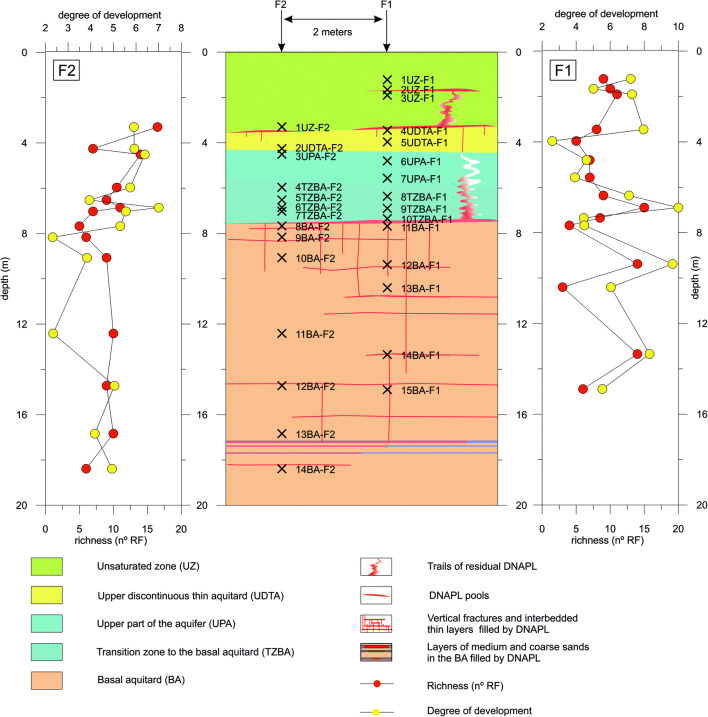
Conceptual model of the source zone. Lateral graphs show richness (no. RF) and degree of development for the F1UB (right side) and the F2UB (left side) boreholes

Microbial richness and the semiquantitative measure of the degree of development indicate hot spots of biogeochemical activity, as well as areas with the inhibition and/or specialisation of some microbial populations. Figure 1 shows the variation in richness and the degree of development in depth and in relation with the hydrostratigraphic units and the distribution of DNAPL. There is a clear correlation between richness and the degree of development in the F1UB borehole, while this correlation is more diffuse in F2UB. The richness and degree of development show relative maximums in the joint points of the BA (12BA-F1 and 14BA-F1) and in the TZBA above the residual pool (9TZBA-F1 and 6TZBA-F2), while the richness shows relative maximums unrelated to the degree of development in the contact areas between the two different hydrostratigraphic units (1UZ-F2 and 3UPA-F2). Generally, the decrease in richness and the degree of development is related to the presence of a high amount of PCE (2UZ-F1, 10TZBA-F1, 11TZBA-F1, 7TZBA-F2, 8BA-F2, and 9BA-F2) and to the hydrostratigraphic units of finer material (5UDTA-F1, 13BA-F1, 15BA-F1, 2UDTA-F2, and 11BA-F2).

## Results

The T-RFLP results for the three restriction enzymes of boreholes F1UB (Figure S1) and F2UB (Figure S2) were highly complex. Within the 29 sediment samples, 40, 70, and 37 different RFs higher than 1% of the total area were detected for MspI, HhaI, and HaeIII, respectively (Figures S1 and S2). For the sediment and groundwater samples, 47, 77, and 45 different RFs for MspI, HhaI, and HaeIII, respectively (Figures S1, S2, and S3), were detected, with at least 8 new RFs in the groundwater. The statistical treatment of cluster analysis (Figures S4, S5, and S6) showed no conclusive results since the samples were grouped in different clusters for each enzyme. Specifically, the microbial communities with higher richness were located in different clusters for each restriction enzyme. The distribution of the samples within each cluster proved that there were some similarities between communities, but none could be explained by its location or a single set of environmental variables. The environmental data tested included the total amount of chloroethene, PCE, TCE, cis dichloroethene (cisDCE), Corg, Mn, and Fe; the depth; and the predominant lithology. There was no dominant variable controlling the composition and distribution of the microbial communities, but there was a group of variables that differed in importance depending on the location. Microbial communities in the presence of contaminants developed in a more complex way given the increased heterogeneity of the medium. Communities developed and had different metabolisms depending on (1) the characteristics of the surface to which they were attached; (2) the balance of nutrients and contaminants between the solid, liquid, and gaseous phases; and (3) the concentration of the pollutants. These cases are referring to heterogeneity on a centimetre scale.

A comparative analysis of the 3 restriction enzymes of each sample allowed the fingerprints of the 10 most abundant populations to be determined (Table 1). These 10 populations were selected since they are the dominant population in at least 1 of the characterised communities. Each set of three RFs was analysed using the PAT (Kent et al. 2003) and double checked with the results of the clone library (Puigserver et al. 2016).

**Table 1:** Most abundant microbial populations, quantified by the restriction fragments (RFs) and identified by the phylogenetic assignment tool (PAT). (+): identified by clone library and PAT. *Identical fingerprint to bacteria in the anaerobic fermentation reactor (GU454879.1.1495), microbial biofilm (DQ499314.1.1492), and groundwater contaminated with nitric acid bearing uranium waste (AY662046.1.1527), among others

**RF**	**HaeIII**	**HhaI**	**MspI**	**Bacteria**	**Phylum**
1	62.5	675	165	*Propionibacterium* acnes (+)	*Actinobacteria*
2	251	205	485	*Acidithiobacillus ferrooxidans*	*γ-Proteobacteria*
3	226	468	160	*Streptomyces, Arthrobacter*	*Actinobacteria*
4	308	585	560	*Streptococcus, Aerococcus viridans* (+)	*Firmicutes* (bacilli)
5	308	236	153	*Aeribacillus pallidus* (+), *Staphylococcus* sp.	*Firmicutes* (bacilli)
6	230	143	279	*Microbacterium* sp., *Terrabacter* sp.	*Actinobacteria*
7	253	207	491	*Acinetobacter junii* (+)	*γ-Proteobacteria*
8	204	363	491	*Haemophilus* sp.	*γ-Proteobacteria*
9	196	204	140	Uncultered bacterium*	
10	217	62	485	*Variovorax paradoxus* (+)	*β-Proteobacteria*

**Fig. 2:**
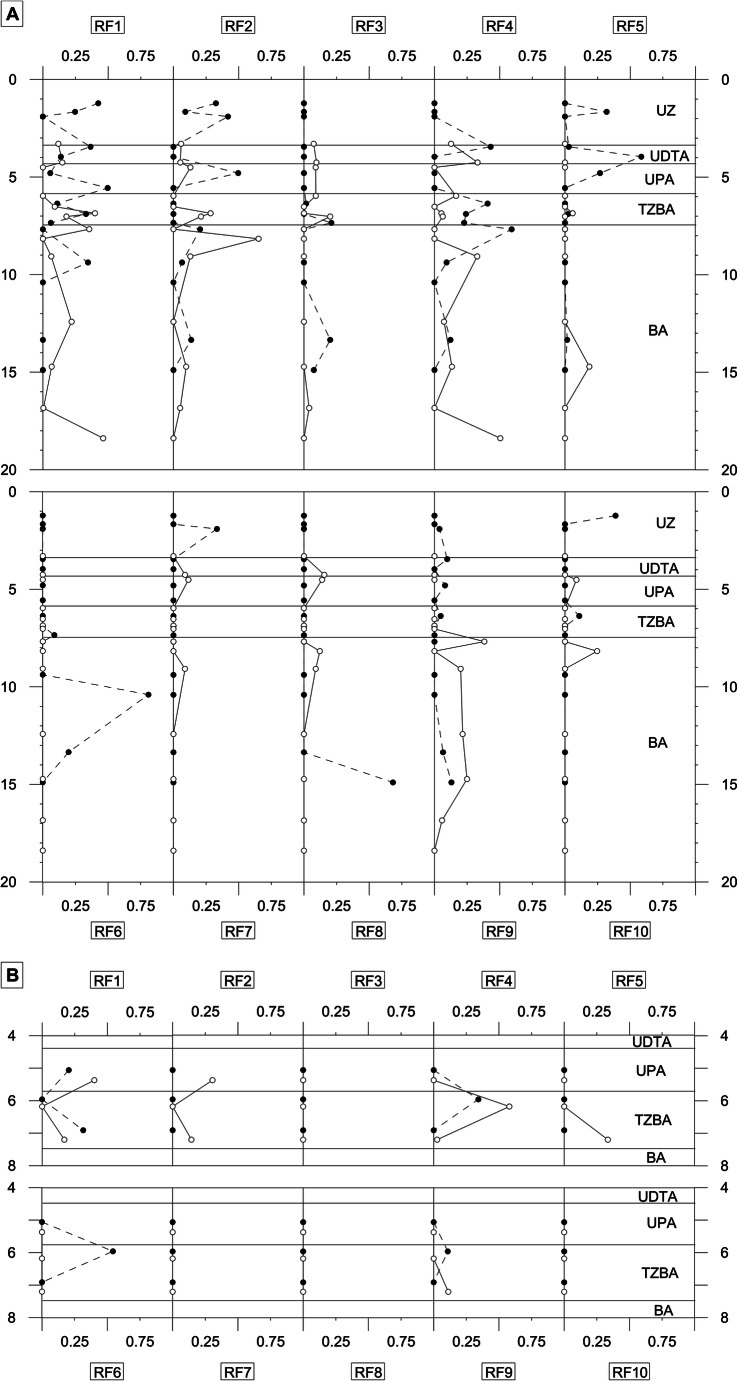
**A** Distribution of the most abundant microbial populations by depth and location of hydrostratigraphic unit. **B** Distribution of the most abundant microbial populations identified in the boreholes, sampled at the ports of multilevel wells F1UB and F2UB. Black dots: F1UB; white dots F2UB

RF1 was identified by the PAT and clone library as *Propionibacterium acnes*, an anaerobic microorganism that produces propionic acid by fermentation (Green 1992) and that is related to the reductive dehalogenation of PCE and TCE (Chang et al. 2011; Moreno et al. 2011). This RF was found almost ubiquitously in the whole study area, in sediments and groundwater, and especially in the contact areas of the different hydrostratigraphic units and the upper and lower levels of the pool of PCE (Figure 2 A and B). RF2 by PAT was identified as *Acidithiobacillus ferrooxidans*, a facultative aerobic organism capable of reducing Fe^3+^ (Ohmura et al. 2002). This RF was distributed heterogeneously along the two boreholes, although it was related to the most oxidant conditions of the upper part of the aquifer and of the unsaturated zone of F1UB (Figure 2A) and to the groundwater of the upper and lower part of the aquifer (Figure 2B). RF3 was identified by PAT as *Streptomyces* sp. or *Arthrobacter* sp. This RF was distributed homogeneously in the UDTA and UPA in the F2UB borehole (Figure 2A). RF4 was identified by PAT as *Streptococcus* sp. and was positively identified by the clone library as *Aerococcus viridans*. The *Aerococcus* genera are microaerophilic (Vela et al. 2007) and autochthonous to groundwater (Cruz-Perez et al. 1996). There was a high proportion of RF4 in the UDTA, the TZBA, and the BA, while it was practically absent from the UZ and the UPA. However, it increased in the TZBA (Figure 2A). Also, RF4 was found in the groundwater at the centre of the aquifer (Figure 2.B). RF5 was identified by PAT as *Staphylococcus* sp. and was positively identified by the clone library as *Aeribacillus pallidus*. RF5 was found in the UZ, the UDTA, and the UPA of F1UB; in the BA of F2UB (Figure 2A); and in the groundwater of the TZBA of F2UB (Figure 2B). RF6 was identified by PAT as *Microbacterium* sp. or *Terrabacter* sp. and was found at the base of the TZBA and the BA of F1UB (Figure 2A) and in the groundwater of the central part of the aquifer in F1UB (Figure 2B). RF7 was identified by PAT and the clone library as *Acinetobacter junii*, an aerobic bacterium that is found ubiquitously in the soil and water and is able to degrade a wide variety of organic compounds (Towner 1992). RF7 was found mainly in the UZ of F1UB, in the interphase of the UDTA and the UPA, and in the upper part of the BA of F2UB (Figure 2A). RF8 was identified by PAT as *Haemophilus* sp. RF8 was detected at the interphase between the UDTA and the UPA, and in the upper part of the BA of the F2UB borehole, and in F1UB at the bottom part of the BA (Figure 2A). RF9 was not identified by PAT and was located mainly in the UDTA of F1UB and the BA of both boreholes (Figure 2A). RF10 was identified by PAT and the clone library as *Variovorax paradoxus*, an aerobic bacterium related to oxidative dehalogenation (Futamata et al. 2005; Humphries et al. 2005). RF10 was located above the peak of PCE of the UZ and the TZBA of F1UB and in the UPA and the BA of F2UB (Figure 2A).

## Discussion

The most abundant RFs (Table 1) were more easily connected to the environmental data since it was possible to identify the main factors determining the distribution of the microbial populations and, therefore, the structure of the microbial communities. These factors were grouped into the following four groups: geological factors (majority granulometry, percentage of fines), hydrogeological factors (capacity to be transported in an aqueous medium), terminal electron-accepting processes (TEAP, e.g. Corg, Mn, Fe, metabolism of the identified populations), and conditioning factors due to the presence of contamination (concentration of PCE and evidence of its degradation).

### Geological factors

**Fig. 3:**
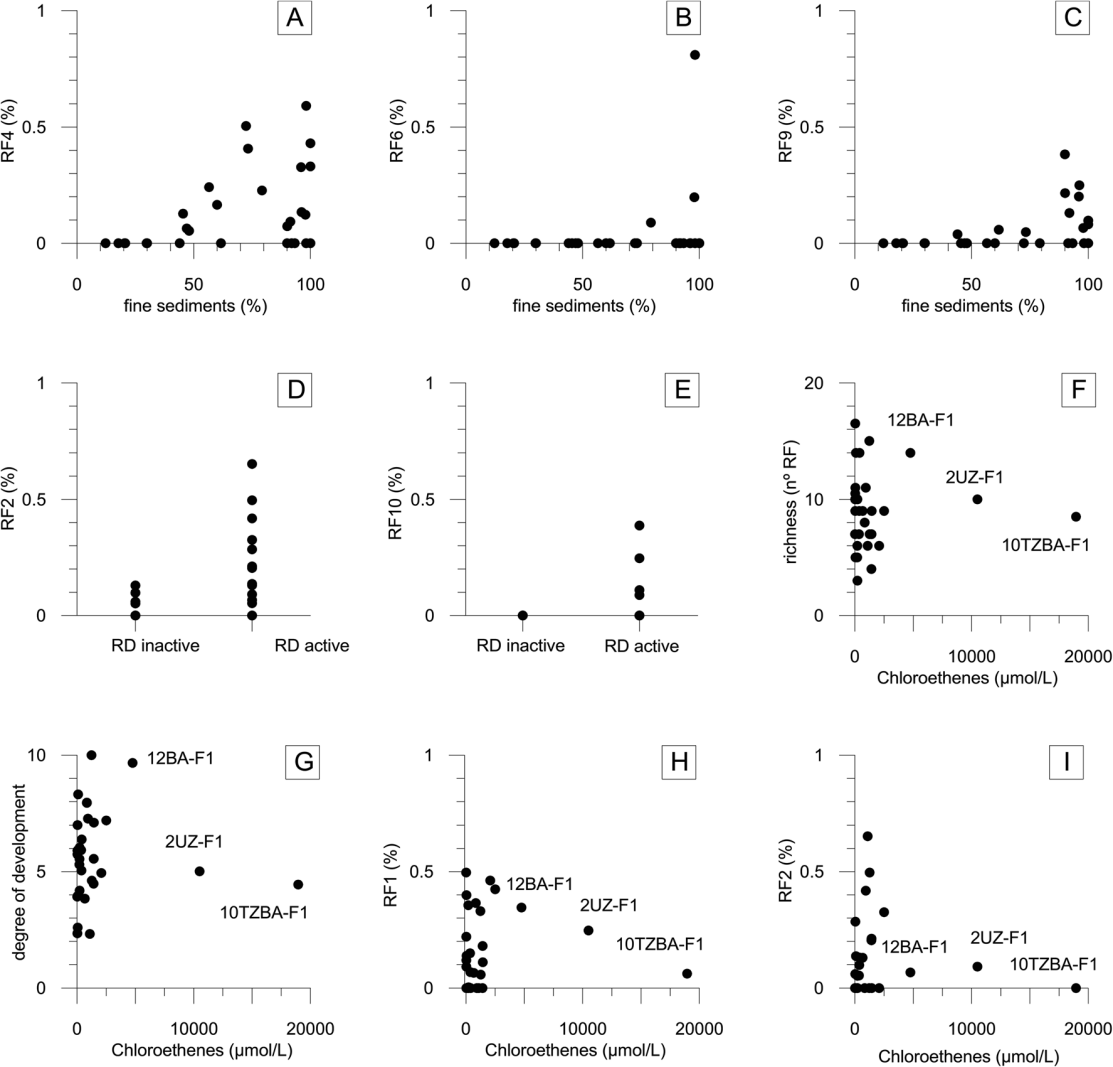
Relationship between the content of fine sediments and RF4 (**A**), RF6 (**B**), and RF9 (**C**). Relationship between reductive dehalogenation processes and RF2 (graph **D**) and RF10 (graph **E**). Relationship between the total sum of chloroethenes (μmol/L) and richness (graph **F**), the degree of development (graph **G**), RF1 (graph **H**), and RF2 (graph **I**). RD reductive dehalogenation

There was a bivariate correlation between the distributions of fine materials, from fine sand to clay (diameter less than 0.25 mm), and certain microorganisms (Figures 3A-C). The RF4, RF6, and RF9 populations were mainly found in the hydrostratigraphic units with more fine materials (UDTA and BA) and in the UPA and TZBA levels with more fine materials (Figure 2). In fact, RF4 and RF9 were not detected in samples with less than 40% of fine materials (Figure 3 A and C), and RF6 was only found in the levels with a minimum of 80% of fine materials (Figure 3B). Other microorganisms, such as RF1, RF2, and RF5, did not show a dependence on sediment granulometry and were distributed throughout the different hydrostratigraphic units (Figure 2).

The absence or very low proportion of specific microbial populations (RF4, RF6, and RF9) in the UZ, the UPA, and the coarser levels of the TZBA, compared to a higher proportion in the finer particle size levels, such as the UDTA and the BA, can be explained in several ways. On the one hand, the finer levels are those with a higher proportion of organic carbon, and the populations may, therefore, be adapted to its degradation (Puigserver et al. 2013). Another explanation could be the non-dependence of the nutrient supply (bioavailability) on saturated sections that are hydraulically more conductive. This would mean that there are populations more capable of taking advantage of the contributions of groundwater (DeAngelis et al. 2010; Meng et al. 2021). Another explanation may be that these populations are not adapted to changes in the physical parameters (such as temperature) or the hydrochemical parameters (such as dissolved oxygen, dissolved organic matter, redox potential, phosphates, and nitrates) of the groundwater (Zhou et al. 2002).

On the other hand, all populations that were identified in the saturated zones (UPA and TZBA) were found in the BA. It can, therefore, be concluded that pore size is not a limiting factor in the distribution of the majority populations. This differs from other studies (Puigserver et al. 2020) that found that pore size limits the colonisation of some bacteria at the finest levels.

### Hydrogeological factors

The ability of bacteria to colonise sediment through the flow of groundwater is another factor that explains the distribution of microbial populations. As can be seen in Figure 2 A and B, RF3, RF7, RF8, and RF10 were only found in sediments and not in groundwater. In the case of RF7 and RF8, this can be explained by their low presence in sediments in the UPA and TZBA, and RF3 and RF10 may not be able to survive in planktonic or floccules form.

The presence of RF1, RF2, and RF5 in the groundwater seems to demonstrate that these microorganisms can colonise other areas of the aquifer, either as floccules, planktonic cells, or attached to clays or silts (Griebler and Lueders 2009). These populations are also related to active biogeochemical processes (denitrification and reduction of Mn and Fe) since they were found in the upper and lower part of the aquifer. These two zones have been defined as ecotones by Herrero et al. (2021a).

The presence of RF4, RF6, and RF9 in the groundwater may be related to whether these bacteria are attached to clays or silts in suspension in the groundwater. On the one hand, these populations are related to the fine materials (previous section), and on the other hand, they were mostly detected in the centre of the aquifer, where no biogeochemical process was detected at a hydrochemical level (Herrero et al. 2021a). In relation to this, Zhao et al. (2012) showed that *Streptococcus* (RF4) was able to adhere to and travel in clay size particles.

### Oxygen tolerance

The tolerance of microorganisms to fluctuating oxygen levels is a limiting factor. The aquifer (UPA and TZBA) had dissolved oxygen concentrations that varied in depth and time (from 12.30 to 0.12 mg/L). Although the medium is generally oxic, there are micro-niches with gradations of oxygen concentration and redox conditions on a millimetre scale, which allow anaerobic microorganisms to metabolise (Rivett et al. 2008; Perović et al. 2017). These gradations are more important when there is more geological heterogeneity, as is the case in the TZBA compared to the UPA (Puigserver et al. 2016). In fact, denitrification and the reduction of Mn were detected in the upper part of the UPA, and denitrification, the reduction of Mn and Fe, and sulphate reduction were detected in the lower part of the TZBA (Herrero et al. 2021a).

Under these conditions, the widely distributed populations of RF1, RF2, RF3, and RF4 in the boreholes were identified as facultative microorganisms. *Propionibacterium acnes* (RF1, Table 1) is mostly considered to be an anaerobic bacterium, although some strains have been identified as facultative or microaerophilic (Stackebrandt et al. 2006). *Acidithiobacillus ferrooxidans* (RF2, Table 1) is a facultative aerobic organism that, in the absence of oxygen, is able to use Fe^3+^ as a final electron acceptor (Ohmura et al. 2002). RF3 was identified as an aerobic bacterium of the genera *Streptomyces* and/or *Arthrobacter*. *Streptomyces* sp. is capable of growing under microaerobic conditions and surviving under anaerobic conditions (Van Keulen et al. 2007), and *Arthrobacter* sp. can grow under anaerobic conditions using fermentation and nitrate ammonification (Eschbach et al. 2003). RF4 was identified as *Streptococcus* sp. and/or *Aerococcus* sp. *Streptococcus* is a facultative organism (Hardie and Whiley 2006), probably derived from agricultural fertilisers that have adapted to the environment (Zhao et al. 2012), and *Aerococcus* sp. is an aerobic facultative organism (Das and Kazy 2014).

In the oxygenated and redox conditions detected, it is possible that biofilms were present, given the capacity of *Propionibacterium* sp. (Tyner and Patel 2016), *Streptomyces* sp. (Liermann et al. 2000), *Terrabacter* sp. (Piazza et al. 2019), and RF10 (DQ499314.1.1492), among others, to produce them. The formation of biofilm would allow a gradient of redox potential and oxygen, which would allow anaerobic microorganisms to have an active metabolism (Davey and O’toole 2000; Aulenta et al. 2006).

### Anaerobic TEAP: reduction of Fe and reductive dehalogenation

The ability of microorganisms to reduce and/or oxidise Mn and Fe is another factor that determines the distribution of microbial populations. The complexity of the processes of the reduction and oxidation of Mn and Fe and the formation of new minerals has not allowed any statistical correlation to be found between any RF and the total Mn and Fe content in the sediment. However, the identification of several populations capable of reducing and/or oxidising these metals is well known. *Acidithiobacillus ferrooxidans* (RF2) oxidises Fe^2+^ under aerobic conditions, and under anaerobic conditions, it is capable of reducing Fe^3+^ (Ohmura et al. 2002). *Terrabacter* sp. (RF6) is related to the ability to oxidise Mn and to microbial communities that oxidise Fe (Piazza et al. 2019). *Staphylococcus* sp. (RF5) and *Arthrobacter* sp. (RF3) have the capacity to reduce Fe^3+^ (Paul et al. 2015).

The reductive dehalogenation of chloroethenes occurs in environments in which there are anaerobic TEAPs (Nijenhuis and Kuntze 2016). The presence or absence of reductive dehalogenation processes can be identified from an increase in metabolic rates (e.g. an increase in TCE with respect to PCE or an increase in cisDCE with respect to TCE [Puigserver et al. 2016]) and the presence of isotopically enriched PCE (Herrero et al. 2021b). The bivariant correlation of RF2 and RF10 with the process of reductive dehalogenation (Figure 3H and I) does not imply that these populations can develop such a process. RF2 (*Acidithiobacillus ferrooxidans*) is related to Fe^3+^ reduction (Ohmura et al. 2002), and RF10 is related to an unidentified bacterium found in an anaerobic bioreactor (GU454879. 1.1495). Consequently, it is assumed that this relationship is due to the more anoxic conditions in which these populations are found.

### Factors arising from the presence of contamination

Toxicity, evaluated via the sum of chloroethenes (CE) in the porewater, was evident for the 10TZBA-F1 (18.900 μmol CE/L) and 2UZ-F1 (10.500 μmol CE/L) samples, was lower in the 12BA-F1 (4.760 μmol CE/L) sample, and was not detected in the other samples, where the concentration was lower than 2.500 μmol CE/L. Toxicity is one variable that decreases microbial diversity and the degree of development (10TZBA-F1 and 2UZ-F1 [Figure 3 F and G] had lower values than the adjacent microbial communities). The same effect was detected in the most abundant populations of the site, RF1 and RF2 (Figure 3 H and I). Decreased diversity resulting from contamination is a consequence of community specialisation (Lima et al. 2018). Some microorganisms (e.g. RF1 and RF2) die because of the poisoning effects of the contaminants, causing the microbial community to transition toward one that is able to withstand contaminants and to even use them in their metabolic pathways.

On the other hand, a relative increase in RF3 was detected in 10TZBA-F1 and in 7TZBA-F2, with the maximums of PCE in the TZBA, and of RF5 in 2UZ-F1, and a maximum of PCE in the UZ (Figure 1). This increase is attributed to the specialisation and absence of the toxicity effect in RF3 and RF5 and to inhibition by toxicity in the other populations.

## Conclusions

The most abundant phylums in the subsoil were *Proteobacteria*, *Actinobacteria*, and *Firmicutes*. The distribution of microbial communities in the sediment in the source zone of chlorinated solvent contamination is highly complex. This distribution can be explained by a group of environmental variables that differ in importance depending on their location, given the high degree of geological and biogeochemical heterogeneity and the complex distribution of the contaminants. Communities develop differently depending on the characteristics of the surface to which they are attached, the biogeochemical conditions of the environment, and the toxicity of the pollutants.

The percentage of fine materials, the capacity of the microorganisms to be transported in an aqueous environment, tolerance to changes in the concentration of dissolved oxygen, capacity to perform TEAP, and toxicity are the factors that were identified as affecting the majority of the populations in this study.

The complexity of the structure of microbial communities in the sediment and the differences from the microbial communities in the groundwater point to the importance of studying these microbial communities when selecting a bioremediation strategy and predicting the response of the microbial communities. Most studies on the effect of chlorinated solvents on microbial communities have relied only on microbial characterisation of the biomass suspended in groundwater, rather than the subsoil. The characterisation of the microbial communities in the two matrices is complementary since the distribution of the populations is different, and populations were only found in one of the two media. Moreover, they allow for better design of bioremediation strategies since environmental factors of the sediment (e.g. geological heterogeneity, Fe minerals, or chloroethenes in the porewater) and limiting factors that may reduce the effectiveness of enhanced reductive dehalogenation (e.g. pore diameter and ability of bacteria to colonise sediment through the flow of groundwater) can be taken into account.

## Supplementary information

ESM 1 (DOCX 5824 kb)

## Data Availability

All data generated or analysed during this study are included in this published article [and its supplementary information files], except for the geochemical data, which could be found in Puigserver et al. (2016), and hydrochemical data, which could be found in Herrero et al. (2021a). The datasets used and analysed during the current study are available from the corresponding author on reasonable request.
