# Supplementary material for: Key factors controlling microbial distribution on a DNAPL source area
Source: Environ Sci Pollut Res Int. 2021 Aug 5;29(1):1508–20. doi: 10.1007/s11356-021-15635-2 (PMC8724114; doi:10.1007/s11356-021-15635-2)
Supplement: Supplementary file 1 — (DOCX 5824 kb) [file 11356_2021_15635_MOESM1_ESM.docx]

**Supplementary information**

Key factors controlling microbial distribution on a DNAPL source area

Jofre Herrero, Diana Puigserver, Ivonne Nijenhuis, Kevin Kuntze and José M. Carmona

| **Sample** | **Nº sample** | **Depth (m)** | **HS unit** | **Borehole** | **Sample** | **Nº sample** | **Depth (m)** | **HS unit** | **Borehole** |
| --- | --- | --- | --- | --- | --- | --- | --- | --- | --- |
| 1UZ-F1 | 1 | 1.22 | UZ | F1 | 1UZ-F2 | 1 | 3.30 | UZ | F2 |
| 2UZ-F1 | 2 | 1.66 | UZ | F1 | 2UDTA-F2 | 2 | 4.25 | UDTA | F2 |
| 3UZ-F1 | 3 | 1.90 | UZ | F1 | 3UPA-F2 | 3 | 4.51 | UPA | F2 |
| 4UDTA-F1 | 4 | 3.45 | UDTA | F1 | 4TZBA-F2 | 4 | 5.97 | TZBA | F2 |
| 5UDTA-F1 | 5 | 3.96 | UDTA | F1 | 5TZBA-F2 | 5 | 6.52 | TZBA | F2 |
| 6UPA-F1 | 6 | 4.80 | UPA | F1 | 6TZBA-F2 | 6 | 6.86 | TZBA | F2 |
| 7UPA-F1 | 7 | 5.57 | UPA | F1 | 7TZBA-F2 | 7 | 7.03 | TZBA | F2 |
| 8TZBA-F1 | 8 | 6.36 | TZBA | F1 | 8BA-F2 | 8 | 7.67 | BA | F2 |
| 9TZBA-F1 | 9 | 6.90 | TZBA | F1 | 9BA-F2 | 9 | 8.16 | BA | F2 |
| 10TZBA-F1 | 10 | 7.35 | TZBA | F1 | 10BA-F2 | 10 | 9.07 | BA | F2 |
| 11BA-F1 | 11 | 7.68 | BA | F1 | 11BA-F2 | 11 | 12.42 | BA | F2 |
| 12BA-F1 | 12 | 9.38 | BA | F1 | 12BA-F2 | 12 | 14.72 | BA | F2 |
| 13BA-F1 | 13 | 10.40 | BA | F1 | 13BA-F2 | 13 | 16.84 | BA | F2 |
| 14BA-F1 | 14 | 13.35 | BA | F1 | 14BA-F2 | 14 | 18.39 | BA | F2 |
| 15BA-F1 | 15 | 14.89 | BA | F1 |  |  |  |  |  |

*Table SI 1: Distribution of the microbial samples. HS: Hydrostratigraphic unit; UZ: unsaturated zone; UDTA: upper discontinuous thin aquitard); UPA: upper part of the aquifer; TZBA: transition zone to the basal aquitard; BA: basal aquitard.*


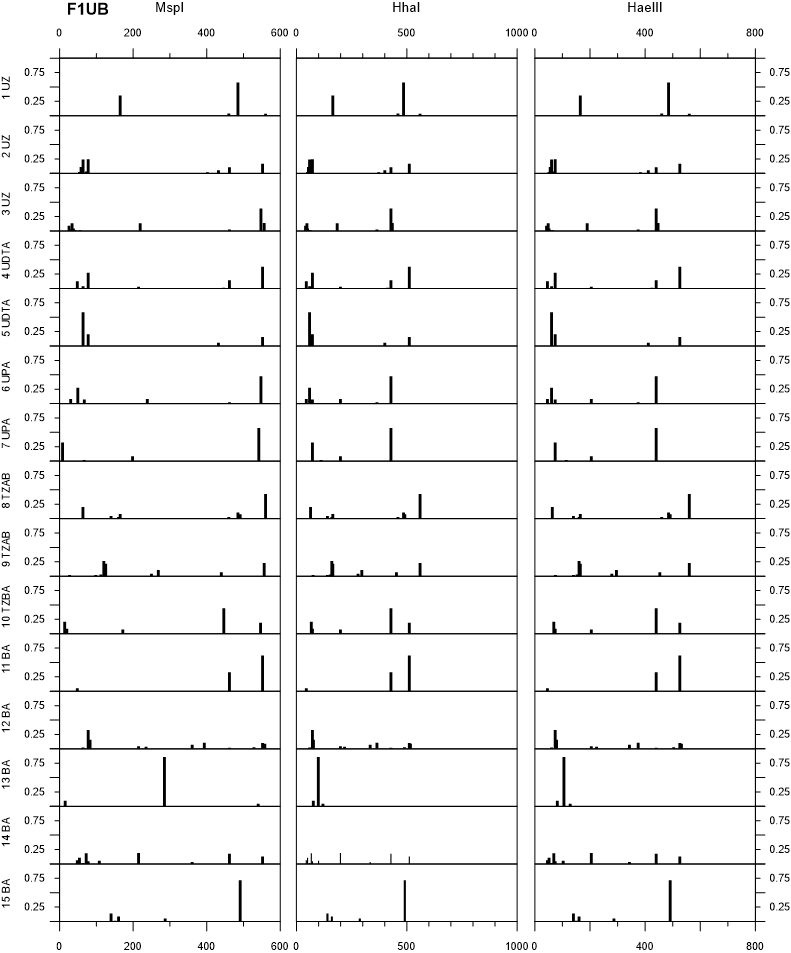


*Figure SI 1: T-RFLP for each sample and restriction enzyme of borehole F1UB. Each graph has standardized proportion (from 0 to 1) and is the average of two valid analysis. X-axis is the length of the RF in base pairs.*


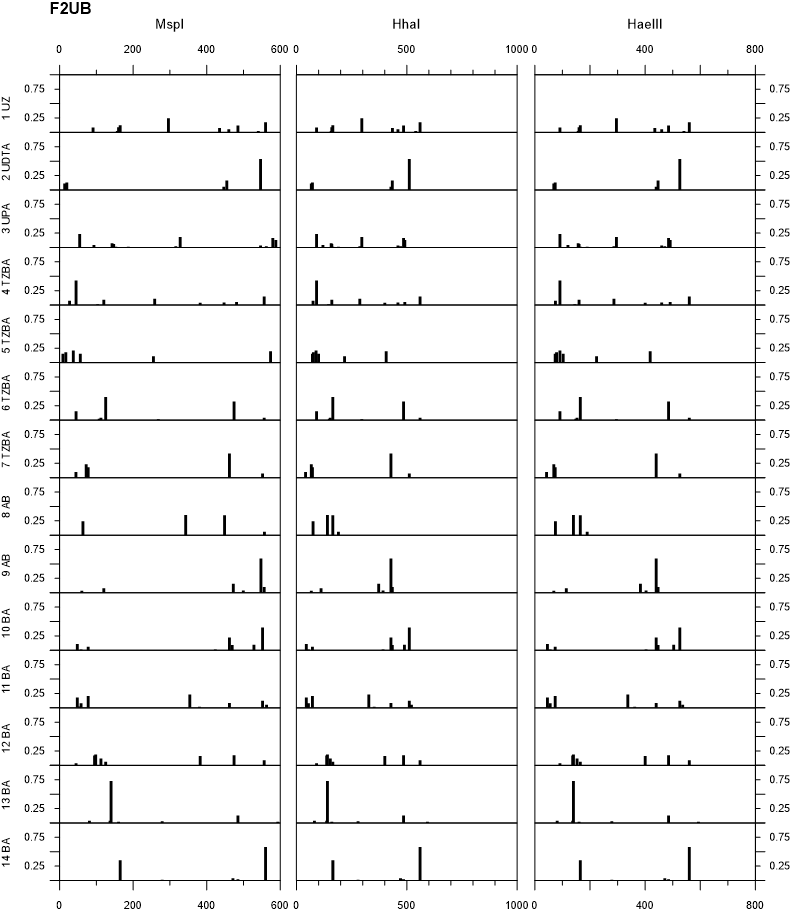


*Figure SI 2: T-RFLP for each sample and restriction enzyme of borehole F2UB. Each graph has standardized proportion (from 0 to 1) and is the average of two valid analysis. X-axis is the length of the RF in base pairs.*


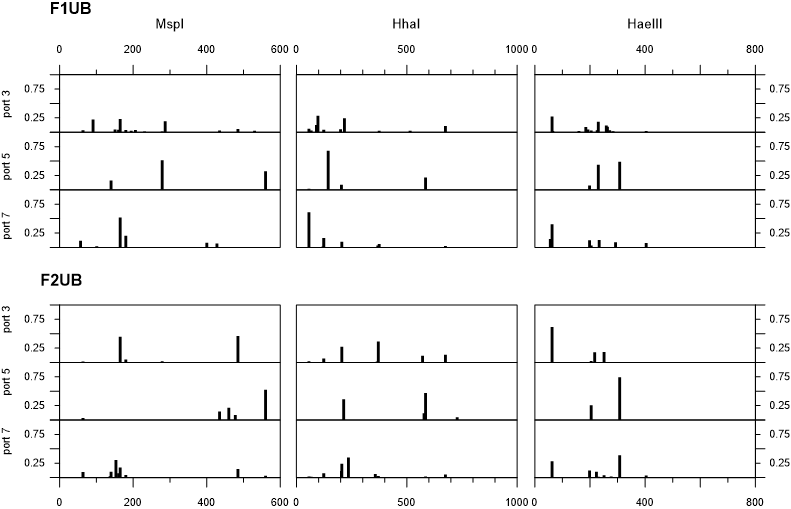


*Figure SI 3: T-RFLP for each sample and restriction enzyme for multilevel wells F1UB and F2UB. Each graph has standardized proportion (from 0 to 1) and is the average of two valid analysis. X-axis is the length of the RF in base pairs.*


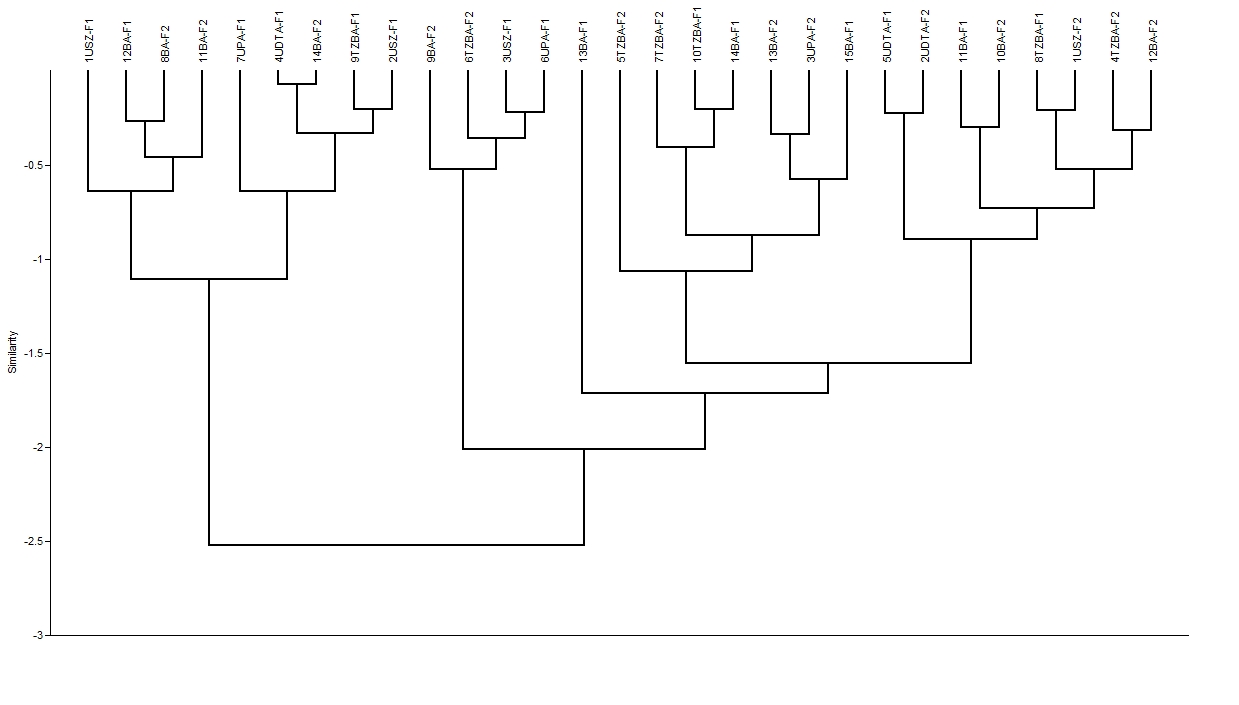


*Figure SI 4: Cluster Analysis with Ward Method of all sediment samples with HaeIII restriction enzyme.*


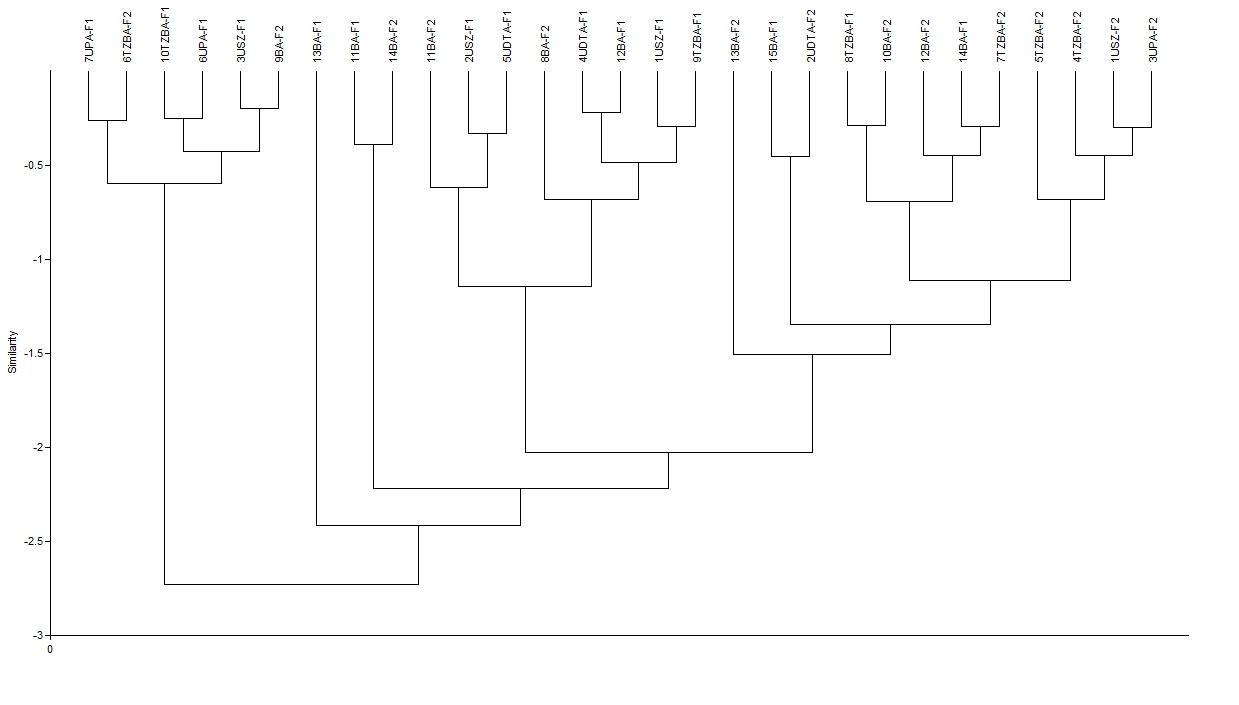


*Figure SI 5: Cluster Analysis with Ward Method of all sediment samples with HhaI restriction enzyme.*


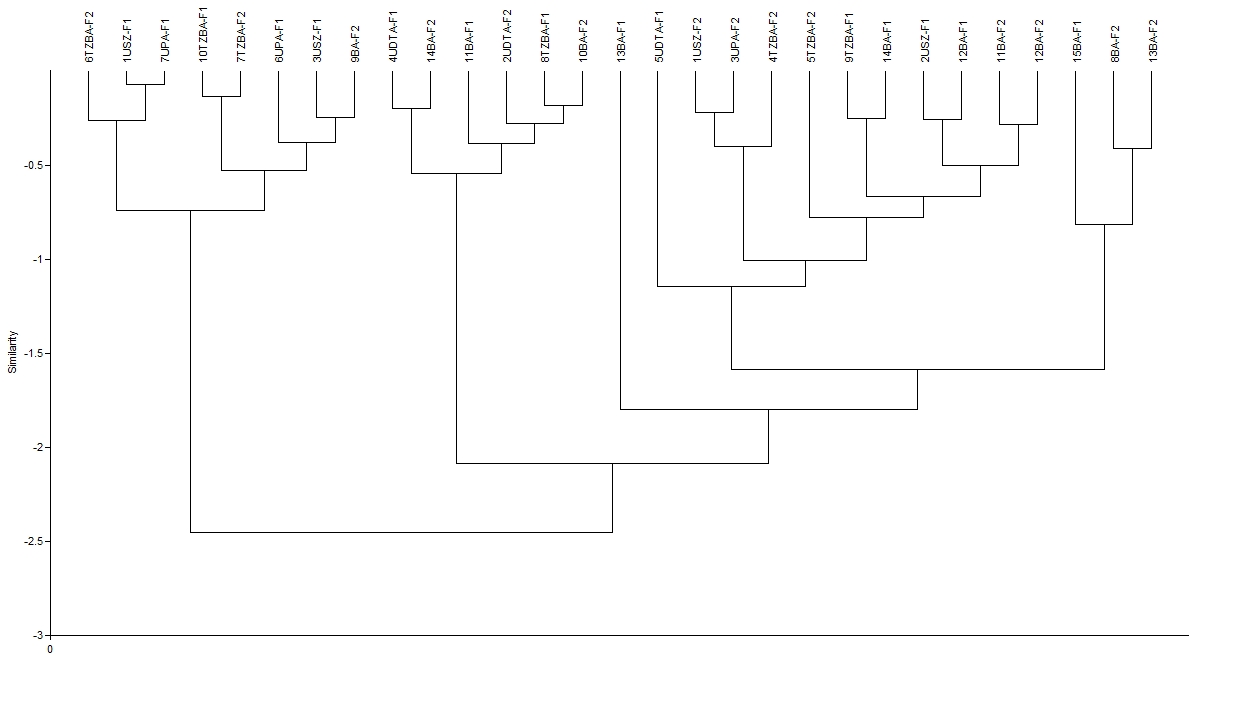


*Figure SI 6: Cluster Analysis with Ward Method of all sediment samples with MspI restriction enzyme.*
